# *C. elegans* show Preference for *Pseudomonas mendocina* (MSPm1) and *Proteus mirabilis* ( *P. mirabilis* sp?) *, and Repulsion to* *Pseudomonas lurida* (MYb11); Growth on *Pseudomonas mendocina* (MSPm1) Increases Attraction to 2-nonanone

**DOI:** 10.17912/micropub.biology.000535

**Published:** 2022-03-17

**Authors:** Alec J Chen, Carlos Zuazo, Katie Mellman, Rashmi Chandra, Noelle L'Etoile

**Affiliations:** 1 Department of Cell and Tissue Biology, University of California, San Francisco; 2 Johns Hopkins Undergraduate

## Abstract

*C. elegans’* experiences and microbiome have been shown to shape its responses to certain stimuli; a recent study found that *C. elegans* grown on *Providencia alcalifaciens* JUb39 exhibited increased attraction to that same growth bacteria while also lowered repulsion to the odor 1-octanol (O’Donnell et al. 2020). This prompted us to ask whether other strains of bacteria could likewise alter *C. elegans’* responses to bacterial food and volatile chemicals. So, to expand upon current knowledge, we cultured wild-type *C. elegans* (N2) on an unidentified *Escherichia coli* ( *E. coli* sp?), *Pseudomonas mendocina* (MSPm1), *Pseudomonas lurida* (MYb11), *Stenotrophomonas maltophilia* (JUb19), or *Proteus mirabilis* strain ( *P. mirabilis* sp?). After several generations, we examined how their choice of bacterial food was affected. In addition, we looked at their response to the olfactory stimuli 2-butanone; 2,3-butanedione; 2,3-pentanedione; and 2-nonanone, as well as their response to the gustatory stimulus sodium chloride. Interestingly, we found that growth on any of these bacterial strains led to their bacterial preferences and behavioral responses to 2-butanone; 2,3-pentanedione; diacetyl; and sodium chloride remaining unchanged. However, we also saw that *C. elegans* showed a preference for MSPm1 and *P. mirabilis* sp? to HB101, and HB101 to MYb11. Furthermore, worms that are grown on MSPm1 showed stronger attraction to a 1:10 dilution of 2-nonanone (AWB-sensed odorant) as compared to worms grown on the other bacterial strains.

**Figure 1. Assessing changes in C. elegans sensory behavior after they were raised on six different strains of bacteria:**
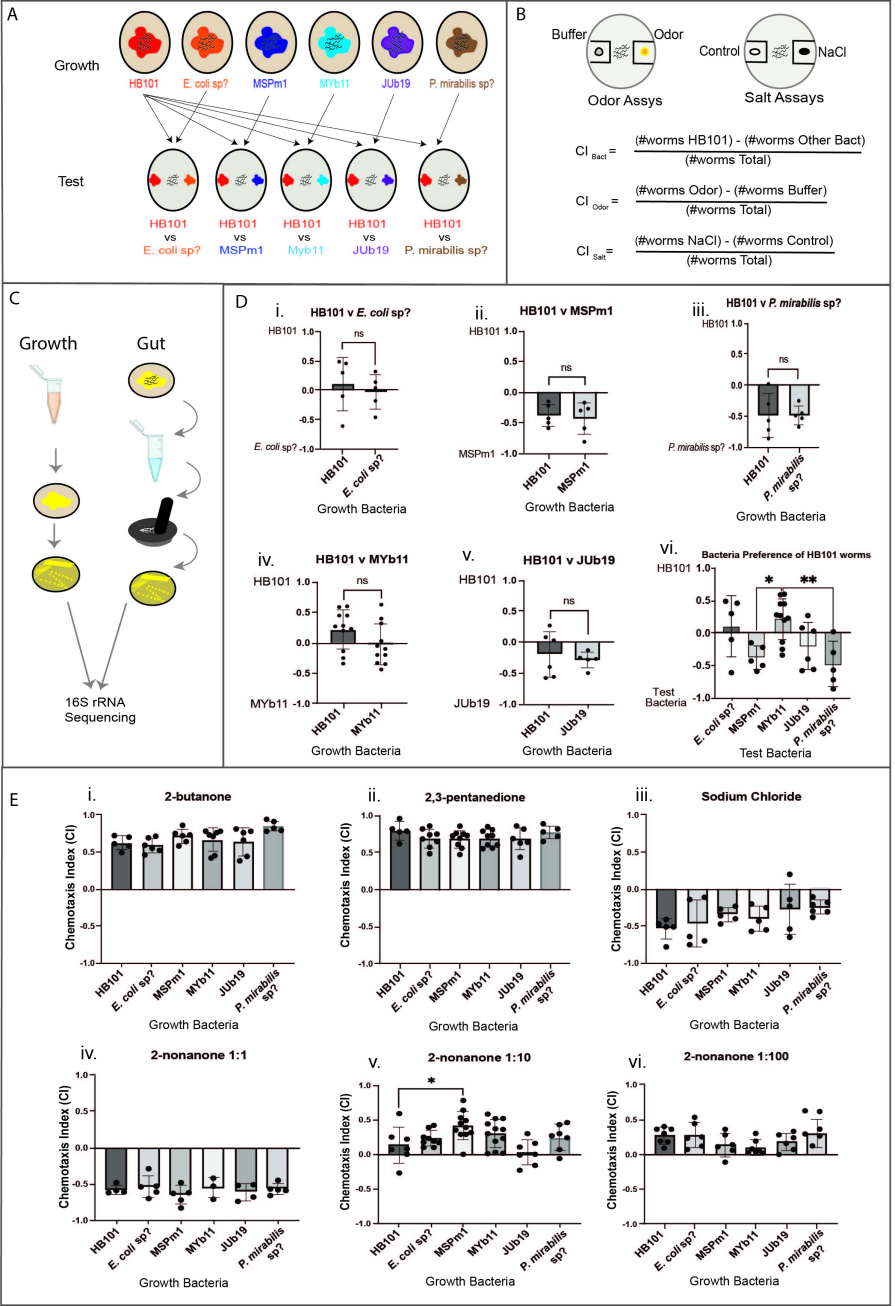
**
A. *C. elegans* Propagation and Bacterial Choice Assays.** The starting generations of N2 animals were bleached free of bacteria and propagated on the specific bacterial strain indicated. After two or more generations, up to eight L4 hermaphrodites were picked onto large NGM plates (10cm radius) with lawns of the same bacterial strains they had previously been grown on. This is labeled as the “Growth” row. Four days later, the day one adult progeny were washed free of bacteria and then assayed for bacterial choice on large chemotaxis plates (distinct from NGM plates) between a choice of two bacterial strains equidistant from the center (where the worms are placed after the washes). Worms raised on HB101 served as a control, and were presented with a choice between HB101 versus every other bacterial strain. Worms raised on the other bacterial strains were presented with a choice between HB101 and their growth bacteria as shown in the “Test” row. **B. Chemical Stimuli Assays and Chemotaxis Index Calculation.** Day one adult progeny (cultured as shown in the “Growth” row from figure 1 panel A) were washed free from their bacterial food and then assayed to determine chemotaxis preference for: 2-butanone; 2,3-butanedione; 2,3-pentanedione; 2-nonanone; and sodium chloride (on large chemotaxis plates). The choice index (CI) formulas are shown. For the bacterial choice assays, the CI equals the number of worms in the HB101 area minus the number of worms in the opposing area then divided by the total number of worms. For the chemical stimuli assays, the CI is determined by the number of worms in the chemical stimuli area minus the number of worms in the ethanol area then divided by the total number of worms. **C. 16S rRNA Sequencing on Growth and Gut Bacteria show the Microbiomes Consist of the Experimentally Provided Growth Bacteria.** From the bacterial lawn in figure 1 panel A, the bacteria were streaked out for single colonies on LB plates. These colonies were then prepared for 16S rRNA sequencing, allowing us to identify the bacteria in the growth medium. This is labeled as the “Growth” column. Under the “Gut” column, we assessed the bacteria in the animal’s intestinal system. The worms were washed clean and then grounded for microbiome analysis: the supernatant of the worm lysis was streaked out onto LB plates, followed by 16S rRNA sequencing on the single colonies. By matching the sequences of both the growth bacteria and gut bacteria, we found that the growth bacteria successfully colonized the worms’ microbiomes. **D. Bacterial Choice Assays Show that the Different *C. elegans* Microbiomes do not Alter their Choice of Bacteria to Feed on and that they Prefer MsPM1 and P. Mirabilis to E. coli HB101.** The chemotaxis indices for the bacteria choice assays with one-way ANOVA significance tests are shown. Comparisons were made between every two groups in graph vi. An index of 1 indicates preference for HB101, while an index of -1 indicates preference for the named bacterial strain. The x-axis shows the growth bacteria of the worms for graphs i-v; in graph vi, the x-axis shows the opposing bacterial strains to HB101 that the HB101-raised worms were tested against (for instance, under the MSPm1 column, the HB101-raised worms were presented with a choice between HB101 and MSPm1). In graph vi, the single level of significance between MSPm1 and MYb11 represents a p-value of 0.0376 and the double level of significance between MYb11 and *P. mirabilis* sp? represents a p-value of 0.0085. **E. Odor and Salt Assay Results Indicate that the *C. elegans* Microbiome only Alters the Animals’ Response to 2-nonanone at 1:10 Dilution.** The chemotaxis indices for the odor and sodium chloride assays with one-way ANOVA significance tests are shown. Comparisons were made between the control group (HB101) and every other group. An index of 1 indicates complete attraction to the odor or salt, while an index of -1 indicates complete repulsion to the odor or salt. The x-axis shows the growth bacteria of the worms. The single level of significance between HB101 and MSPm1 represents a p-value of 0.0219.

## Description

Diet is one of the main factors that affects the *C. elegans* gut microbiota (Berg et al. 2016). The volatile chemical 1-octanol is repulsive to worms grown on *E. coli.* , however, O’Donnell et al *.* (2020) showed that when *C. elegans* are grown on *P. providencia* , the tyramine produced by *P. providencia* increases the endogenous octopamine levels in the worms, which then affects the ASH-mediated responses such that the ASH-meditaded repulsion from 1-octanol is diminished. Additionally, this study showed that repulsion to 2-nonanone, an AWB-mediated response (Fekerey et al. 2021), was not affected by growth on *P. providencia* . Though these studies have examined how growth on non-traditional bacterial strains affect chemosensory responses (Berg et al. 2016 and O’Donnell et al. 2020), most studies have and continue to propagate *C. elegans* on various strains of *E. coli* . Thus, there is a gap in our understanding of how growth bacteria influences the nervous system responses of *C. elegans* .

We hypothesized that growth on different bacterial strains would change the behavioral responses of *C. elegans* as compared to the responses of worms grown on *E. coli.* Firstly, we explored the influence of growth bacteria on their food choice by growing worms on a given bacterial strain for two or more generations before presenting them a bacterial choice assay (Figure 1 panel A). It is important to note that we verified that the bacteria they were grown on actually colonized the gut of the worms by performing 16S rRNA sequencing of bacteria isolated from the gut of *C. elegans* . We adapted the protocol from Berg et al. (2016) (Figure 1 panel C). Based on the CIs (to measure preference) in figure 1 panel D, graph i indicates that worms raised on HB101 and *E. coli* sp? both showed equal attraction to HB101 and *E. coli* sp?, graph ii indicates that worms raised on HB101 and MSPm1 both showed preference for MSPm1 over HB101, graph iii indicates that worms raised on HB101 and *P. Mirabilis* sp? both showed preference for *P. Mirabilis* sp? over HB101, graph iv indicates that worms raised on HB101 and MYb11 both showed equal attraction to HB101 and MYb11, and graph v indicates that worms raised on HB101 and JUb19 both showed equal attraction to HB101 and JUb19. Interestingly, graph vi shows that HB101-raised worms were seemingly more attracted to MSPm1 and *P. mirabilis* sp? than MYb11, however, it does not translate to *C. elegans* preferring MSPm1 or *P. mirabilis* sp? over MYb11 based on a finding that bacterial preference is not transitive (Iwanir et al. 2019). Put together, our results conclude that no matter the makeup of the worms’ gut microbiome, they preferred MSPm1 and *P. Mirabilis* sp? over HB101 (Figure 1 panel D graphs ii, iii, vi). Consequently, it demonstrates that when the worms’ microbiomes consisted of these bacteria, it had no effect on their bacterial choices.

In *C. elegans* , the AWC, AWA, AWB, ASE/L, and ASE/R chemosensory neurons were all shown to respond with changes in calcium levels when presented with *E. Coli* (Zaslaver *et al.* 2015). This sparked our interest in exploring whether the microbiome affects the behavioral responses driven by sensory neurons. We chose to study responses elicited by chemical stimuli that act through known sensory neurons (Ferkey et al., 2021). AWC ^on^ detects 2-butanone; AWC ^off^ detects 2,3-pentanedione; AWB detects 2-nonanone; and both ASE/L and ASE/R detect sodium chloride (Ferkey et al *.* 2021; Woldemariam et al. 2019). Using the chemotaxis assay depicted in Figure 1B to quantify a CI value, we found that there was no change in attraction to 2-butanone (Figure 1 pane E graph i) or 2,3-pentanedione (Figure 1 panel E graph ii), and no change in in repulsion from sodium chloride (Figure 1 panel E graph iii) as a function of microbiome. However, animals that had an MSPm1 microbiome displayed an attraction to 2-nonanone at a 1:10 concentration that was significantly greater than animals with HB101 microbiome (Figure 1 panel E graph v).

**Discussion:**

We had asked whether the *C. elegans* microbiome alters food choice and we found that none of the bacterial strains used in this study did. HB101 was chosen over the more common OP50 strain because raising worms on HB101 has been shown to be more nutritious (Neve et al. 2020). Since HB101-raised worms showed CIs away from the maximum or minimum indices, any increase or decrease in attraction to the test bacteria (in the worms raised on the other bacterial strains) would have been readily observable in our assays. However, growth on bacteria that are slightly more attractive to HB101-raised worms, such as MspM1 or *P. mirabilis* sp?, did not cause the animals to be more attracted to either of these bacteria. Likewise, growth on MYb11, the slightly less attractive strain, also did not alter the behavior of the worms. This may indicate that the neural responses that are associated with the preferences seen in HB101-raised animals cannot be changed by the worms’ microbiome. Given the bacterial strains we used seem to promote healthy propagation (much like HB101) and not be pathogenic, like *P. aeruginosa* (Worthy et al. 2018), the worms may just show an indifference to their microbiome, thus, showing no change in their food preferences. Perhaps if we had examined bacterial strains that provided either a more positive or negative experience for the worms than HB101 does, we would have observed a significant change in preference. Previous studies have shown that growth on pathogenic bacteria will lead to repulsion from that strain of bacteria (Zhang et al. 2005). Also, growth on *E. coli* that express dsRNA targeting key metabolic processes will cause these animals to be repulsed from lawns of *E. coli* (Melo and Ruvkun 2012). Therefore, in future studies, we could investigate whether more nutritious bacteria (Honda et al. 2006) would be preferred. Though there was precedent for the microbiome to affect bacterial preference, we did not find that in our study with these five non-traditional bacterial growth strains.

Since we saw that the *C. elegans* microbiome did not affect food choice, we were led to ask whether it would alter chemosensory responses. Interestingly, we found that raising *C. elegans* on MSPm1 creates a slight alteration in attraction to 2-nonanone, specifically that it will increase their peak attraction to this volatile chemical at a 1:10 dilution. This finding is similar to that of O'Donnell et al. (2016), in which they found that the repulsive odor 1-octanol became less repulsive after growth on *P. providencia* . The O’Donnell study found that the neurotransmitter response of the ASH sensory neuron was altered as this neuron is responsible for 1-octanol avoidance. Based on this finding, we propose the idea that the 2-nonanone responsive AWB neuron could have altered neurotransmitter responses when grown on MSPm1-raised worms as compared to HB101-raised worms. If so, this would indicate that different bacteria alter their host’s sensory responses in similar fashion.

Our results with 2-nonanone are also interesting in another facet. The 1:10 and 1:100 dilutions showed attraction while the 1:1 dilution showed repulsion from the worms. This contradicts a previous study which showed that worms were repulsed from a 1:10 dilution of 2-nonanone (Troemel et al. 1997). An important distinction is that that study used avoidance assays on square plates while our study utilized attraction assays on circular plates, which could be a possible explanation for the difference. Nonetheless, the worms were all presented with identical experimental settings so comparing the results still gives a reliable measure of the effects of the different growth bacteria.

Given that the growth bacteria affects certain behavioral choices of *C. elegans* , there are implications for many past and future studies due to the possibility of the role that an *E. coli* diet might play in their results. Future directions will need to implement neural imaging to study the exact way different microbiome backgrounds affect the nervous system responses of *C. elegans* .

## Methods

- N2 worms were propagated on nematode growth media (NGM) plates under standard conditions (Brenner 1974). The plates were seeded with their respective bacterial strains. Worms were kept at RT (21°C). Worm colonies were chunked or picked onto new plates every one or two days to prevent starvation.

- Up to eight L4 worms were picked onto new NGM plates with the same respective bacterial lawn. Four days later, the day one adult progeny were used for behavior assays.

- Each bacterial strain was streaked for single colonies on a fresh LB plate and grown at 30°C for two days. A single colony of each strain was inoculated into 100mL of LB in a 250ml glass bottle and incubated at 30°C overnight without shaking. Large NGM plates (10cm radius) were seeded with approximately 100 microliters of the overnight bacterial culture and then incubated at 30°C for 2 days. Afterward, the seeded plates were stored at RT for up to 2 weeks. Plates that had been equilibrated to RT for 2 days were used in worm culture.

- Our gut microbiome bacteria isolation protocol was adapted from the work of *Berg* *et al.* (2016). Worms were washed off NGM plates in S. basal and collected in 1.5mL tubes, then washed in M9 buffer 5 times. Worms were then grounded using a mortar and pesto. The supernatant was then streaked onto an LB plate and incubated at 30° C. After two days, the bacteria was sent off for 16S rRNA sequencing.

- Our bacteria choice assays were adapted from the work of Glater *et al* . (2014). Each strain of bacteria was grown overnight in LB at 30° C and then spun down in a centrifuge for 10 minutes at 4700 rpm and 20° C. The bacteria was then resuspended in fresh LB at an OD600 of 10 and kept at 4° C for up to a week. We poured chemotaxis plates by first boiling 3.2g of agarose in 200mL of ddH _2_ O until no visible granules, then adding 1mL 1M K _3_ PO _4_ , 200μL 1M CaCl _2_ , and 200μL 1M MgSO _4_ once it cooled to 65° C, and then pipetted 10mL of the gel per petri dish. Once the gel was cooled to room temperature, 25μL of each corresponding bacteria was seeded onto chemotaxis plates and allowed to dry with the lids closed for 16 hours. The animals were washed off the NGM plates with S. basal (5-8mL per plate), transferred to 1.5mL tubes, washed again with S. basal, then washed a final time in ddH _2_ O, before being plated (50-300 worms per plate) onto the chemotaxis plates with the origin at the center. Worms were gently dabbed dry with a Kimtech wipe. 1μL of 1M sodium azide was added to the center of each bacteria spot one hour later.

- We first poured the chemotaxis plates (same recipe as in the bacteria choice assays). 1μL of the diluted odor was added to one spot and 1μL of ethanol was added in the opposite spot, along with 1μL of 1M sodium azide. 2-butanone was diluted 1:1,000 in ethanol. 2,3-pentanedione was diluted 1:10,000 in ethanol. 2-nonanone was diluted 1:1, 1:10, and 1:100 in ethanol. Worms were then washed and plated (same method as in the bacteria choice assays).

- Our sodium chloride assays were adapted from the work of Woldemariam *et al.* (2019). We made the 150mM and 0mM salt plugs by boiling 2g of agarose in 94.3mL (for 150mM) and 97.3mL (for 0mM) of ddH _2_ O until no visible granules, then added 2.5mL 1M K _3_ PO _4_ , 200μL 1M CaCl _2_ , 200μL 1M MgSO _4_ , and 3mL 5M NaCl once it cooled to 65° C, and then pipetted 30mL of the gel per petri dish. Once the plates cooled to room temperature, we used the size 9 cork borer to make the plugs. We made the chemotaxis plates by boiling 2g of agarose in 96.3mL of ddH _2_ O until no visible granules, then added 2.5mL 1M K _3_ PO _4_ , 200μL 1M CaCl _2_ , 200μL 1M MgSO _4_ , and 1mL 5M NaCl once it cooled to 65° C, and then pipetted 10mL of the gel per petri dish. Once the chemotaxis plates cooled to room temperature, we placed the plugs at the opposite sides of the plates and stored the plates at 20° C for 16 hours. Once the plugs were removed, we added 1μL of 1M sodium azide to each spot. Then the worms were washed and plated (same method as in the bacteria choice assays).

- Statistics were performed using Prism GraphPad 8. P values used for the statistical readouts are ns is P > 0.05, *P < 0.05, **P < 0.01, ***P < 0.001, and ****P < 0.0001. N for each data represents the number of independent trials performed for each experiment and are shown as black dots (minimum of n=5 for every bar on each graph). Each independent trial (assay) was performed on a population of >50 worms, and were picked and raised on different growth plates. In panel D, paired t-tests were performed to compare the differences between two groups of data. One-way ANOVA with Bonferroni’s correction reported the statistical differences in the graphs that have more than two groups compared at the same time.

## Reagents

**Table:** 

Reagent Type	Name	Available From	Species/Sequence
Worm Strain	N2	CGC	*Caenorhabditis elegans*
Bacterial Strain	HB101	CGC	*Escherichia coli*
Bacterial Strain	*E. coli* sp?	This study	*Escherichia coli*
Bacterial Strain	MSPm1	CGC	*Pseudomonas mendocina*
Bacterial Strain	MYb11	CGC	*Pseudomonas lurida*
Bacterial Strain	JUb19	CGC	*Stenotrophomonas maltophilia*
Bacterial Strain	*P. mirabilis* sp?	This study	*Proteus mirabilis*
PCR Primer	27F	ELIM Biopharmaceuticals, Inc.	AGA GTT TGA TCM TGG CTC AG
PCR Primer	1492R	ELIM Biopharmaceuticals, Inc.	GGT TAC CTT GTT ACG ACT T
DNA Sequencing Primer	515F	ELIM Biopharmaceuticals, Inc.	GTG CCA GCM GCC GCG GTA A
DNA Sequencing Primer	911R	ELIM Biopharmaceuticals, Inc.	GCC CCC GTC AAT TCM TTT GA
DNA Sequencing Primer	1391R	ELIM Biopharmaceuticals, Inc.	GAC GGG CGG TGT GTR CA
